# Pivotal Surgical Intervention for Pediatric Ileoileal Intussusception With Meckel’s Diverticulum: A Case Report

**DOI:** 10.7759/cureus.60073

**Published:** 2024-05-10

**Authors:** Dheeraj Surya, Pankaj Gharde, Kavyanjali Reddy, Raju K Shinde, Harshal Tayade, Mihir Patil

**Affiliations:** 1 General Surgery, Jawaharlal Nehru Medical College, Datta Meghe Institute of Higher Education & Research, Wardha, IND

**Keywords:** emergency abdominal surgery, emergency exploratory laparotomy, pediatrics emergency, ileo-ileal intussusception, meckel´s diverticulum

## Abstract

The most prevalent congenital gastrointestinal tract abnormality is Meckel’s diverticulum. It is discovered in most instances incidentally. It can be observed as painless bleeding in the gastrointestinal tract. However, it can occasionally result in acute intestinal obstruction, which frequently masks the actual clinical presentation. This is a case of a four-and-a-half-year-old male child who presented with features of obstruction, which, on further evaluation, revealed ileoileal intussusception. An emergency surgical intervention was planned with an exploratory laparotomy and a reduction of intussusception. This case emphasizes the urgency of diagnosing and managing intussusception to prevent serious consequences such as bowel ischemia, bowel necrosis, bowel perforation, peritonitis, and sepsis. It stands as a stark reminder for medical professionals to stay vigilant for these critical gastrointestinal emergencies, and immediate treatment with a multidisciplinary approach is recommended to significantly enhance patient outcomes.

## Introduction

Meckel’s diverticulum (MD), despite being a common gastrointestinal defect, does not usually have a symptomatic presentation. Its symptoms indicate the presence of an underlying disease process and point toward potential MD consequences, including bleeding, obstructed intestines, inflammation, and intussusception [1,2]. When the diverticulum turns inward, it is known as an inverted diverticulum. This uncommon condition is seldom encountered in clinical settings, with only a few cases documented so far [3]. The “rule of twos” has been used to describe this congenital defect. It is commonly located at two feet proximal to the ileocecal junction, and the incidence is likely two times higher in males, present before the age of two, and affects nearly 2% of the population [4]. MD is the true diverticulum, which contains all the layers of the bowel. MD is largely silent, especially in adults. Among the risk factors known to induce symptomatic MD are male gender, age less than 50, the presence of a diverticulum 2 cm or larger in length, or heterotopic mucosa [5]. Failure in the proper closure of the omphalomesenteric duct during fetal development results in the formation of this diverticulum [6]. Ectopic gastric mucosa might cause discomfort due to abdominal pain and gastrointestinal bleeding. Most instances remain asymptomatic throughout life, with their frequency in the general population estimated to be between 0.3% and 2.9%. Inflammation with or without intestinal perforation, gastrointestinal bleeding, and intestinal obstruction are the most commonly observed symptoms [7]. Diagnosis can be more challenging in instances with atypical presentations that include only gastrointestinal symptoms other than blood-containing stools. Although cases of Meckel’s without symptoms can also be presented, roughly 30% of the patients show clinical symptoms at some point in their lives [8]. Thus, despite a very long history of the disease, there are very few examples of children experiencing vomiting and stomach pain without bloody stools, which does not require emergency surgical intervention for ileoileal intussusception [9]. MD has also been associated as a risk factor due to the occasional inversion for ileoileal intussusception in both children and adults [10].

## Case presentation

**Figure 1:**
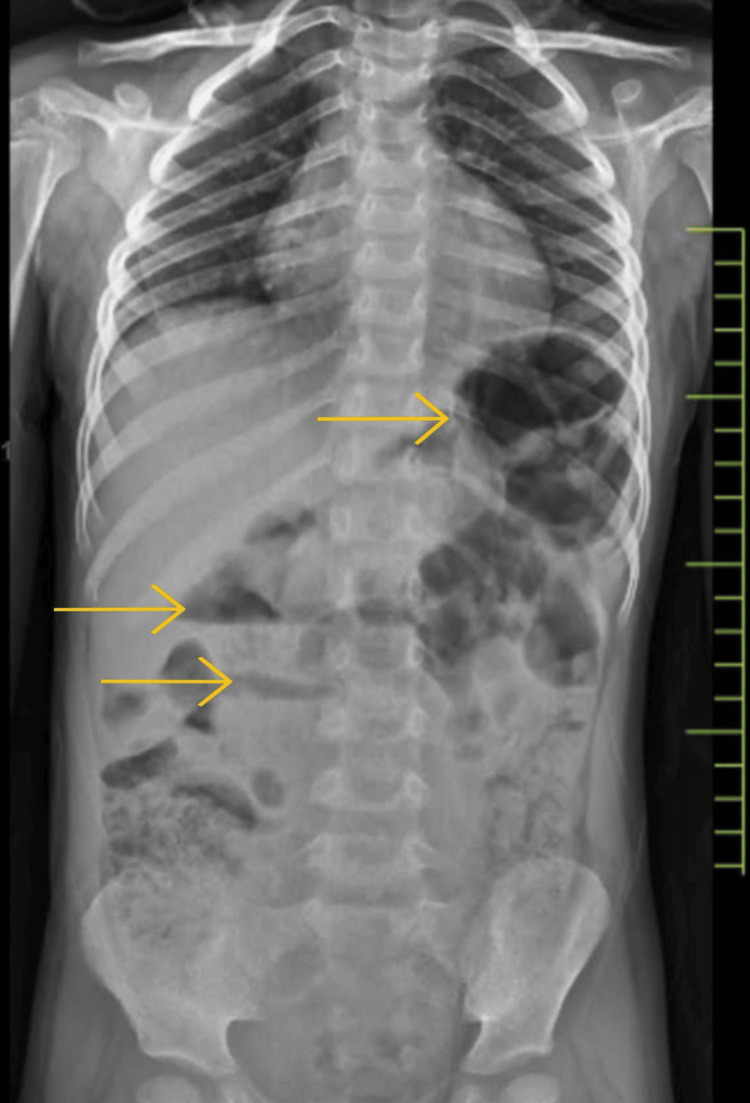
Abdominal X-ray showing multiple air-fluid levels (marked with yellow arrows) ominously scattered

We present the case of a four-and-a-half-year-old male child who was admitted to our hospital with major complaints of abdominal distension, pain, and vomiting for three days. He was vitally stable and afebrile at the time of admission and did not have any significant past medical or family history. On further examination, his abdomen was tender and distended. On auscultation, there were exaggerated bowel sounds. There was no palpable organomegaly observed on admission. The hemoglobin level was 6.9 grams/deciliter, with a peripheral smear showing hypochromic microcytic red blood cells and a serum sodium level of 126 milliequivalents per liter. The rest of the blood parameters were found to be within normal limits. An X-ray of the abdomen revealed multiple air-fluid levels, a distended small bowl, an empty rectum, and no air under the diaphragm (Figure 1).

**Figure 2:**
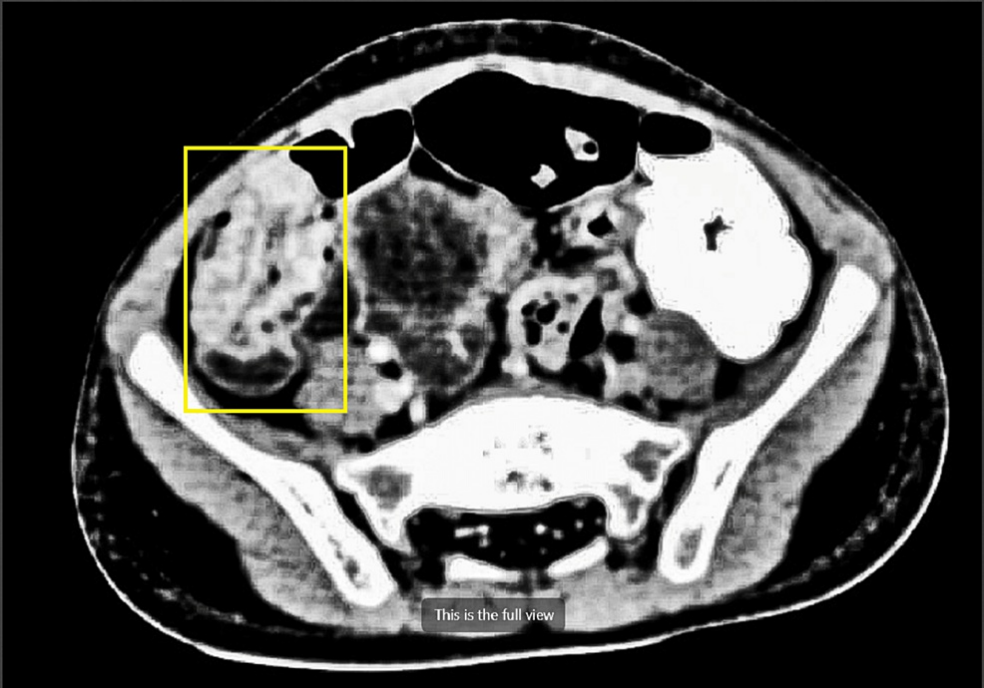
Contrast computerized tomography image of the right ileac fossa – bowel telescoped within itself, showing intussusception (marked with a yellow box)

Ultrasound imaging of the abdomen showed a positive “target sign,” and contrast-enhanced computed tomography imaging of the abdomen was found to be suggestive of a short segment of bowel within the bowel seen in the right iliac fossa involving the ilea’s loops, suggesting intussusception (Figure 2).

**Figure 3:**
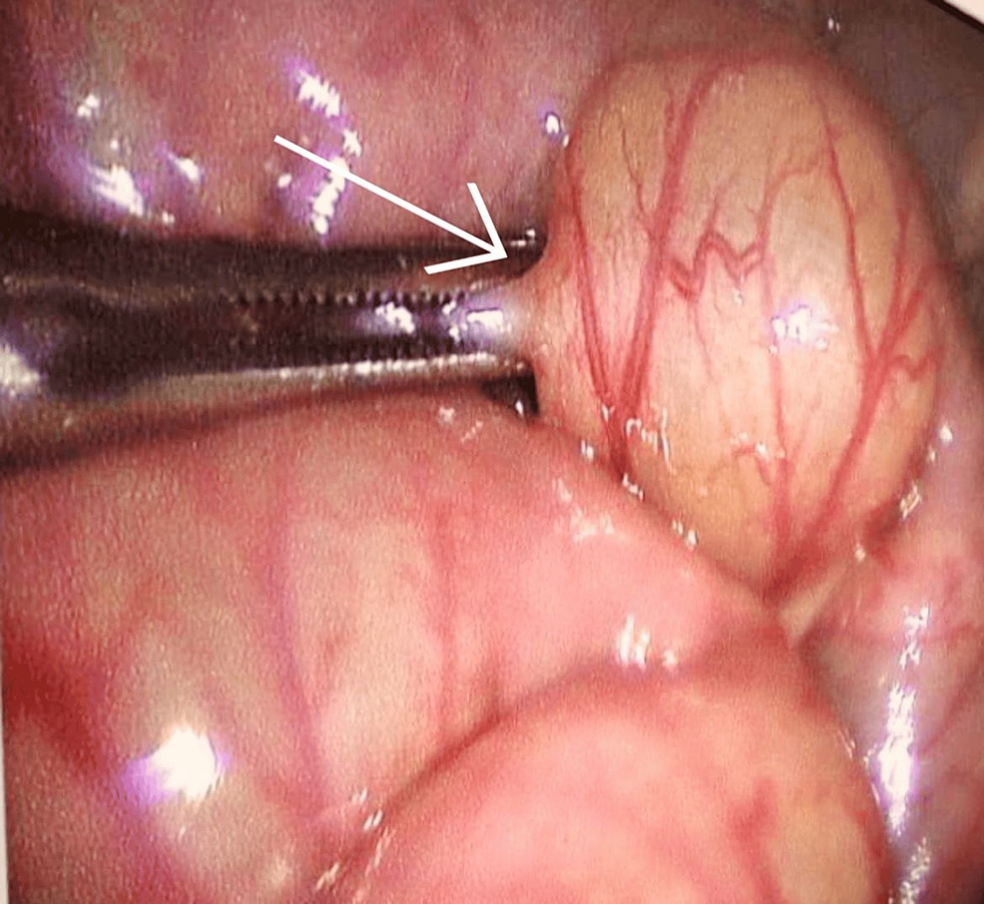
A laparoscopic image reveals severely distended and tangled bowel loops (marked with a white arrow)

**Figure 4:**
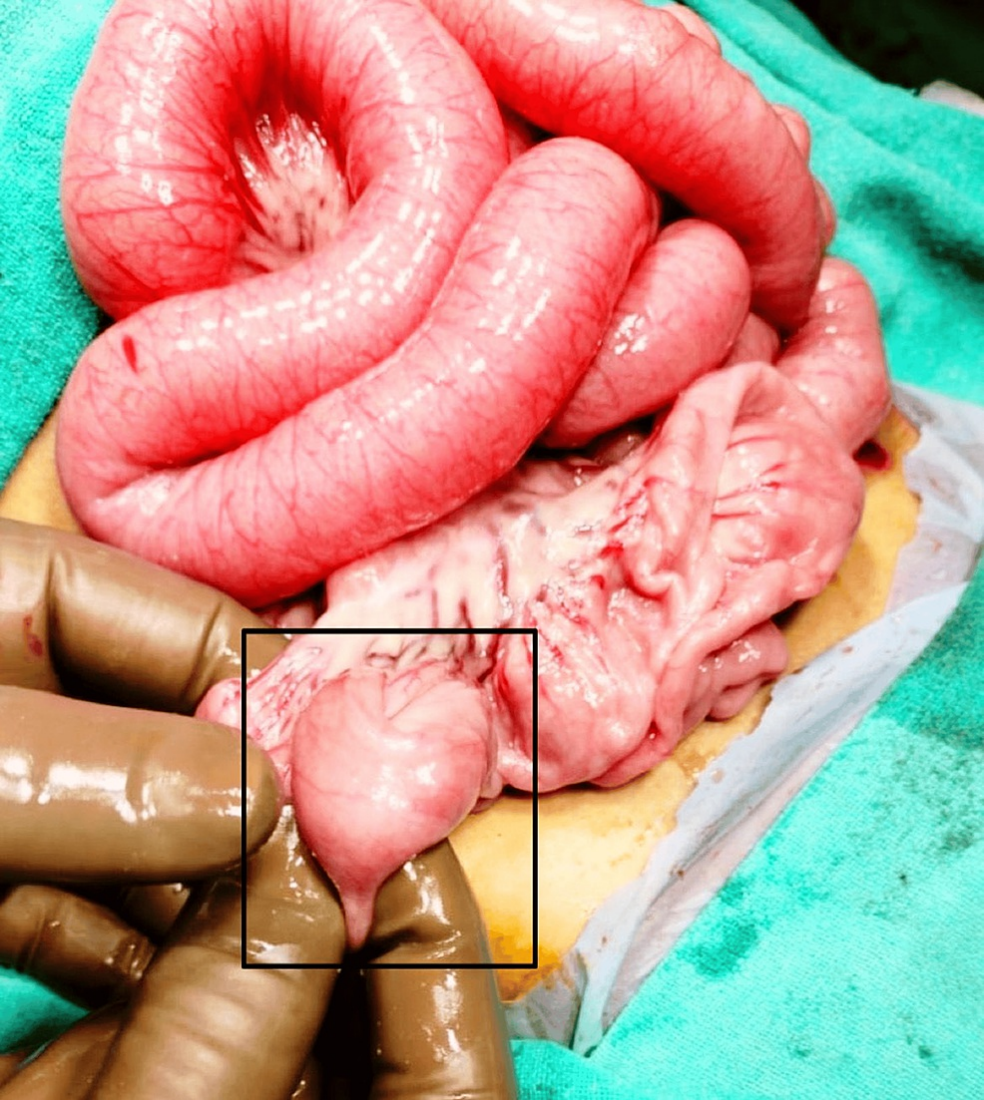
Zone of intussusception in the proximal region of the ileocaecal junction (marked with a black box)

However, no significant proximal small bowel dilatation was observed. The colon was dilated and fecal-loaded. The patient’s condition improved after being kept nil by mouth and receiving intravenous hydration. The procedure was started by nasogastric tube insertion, followed by per rectal stimulation for five minutes continuously, which resulted in relief of distension as the patient passed the residual stool present in the distal bowel. A repeat ultrasonography was done, which revealed the persistence of intussusception. Hence, the decision was made to perform a laparotomy. A preanesthetic workup was done under all aseptic conditions after the instillation of general anesthesia and routine procedures, followed by two ports inserted: the first umbilical port and a second suprapubic port. Intraoperative findings revealed distended proximal small bowel loops, compressed distal bowel loops, and a zone of intussusception noticed in the ileum. The procedure was converted to open surgery, with an incision extended laterally from the umbilical incision. The incision deepened. The subcutaneous tissue was dissected, and the rectus sheath was opened. The peritoneum was identified and opened. The bowel was examined, and ileoileal intussusception was identified approximately 40 cm from the ileocolic junction over the proximal ileum (Figure 3, Figure 4).

**Figure 5:**
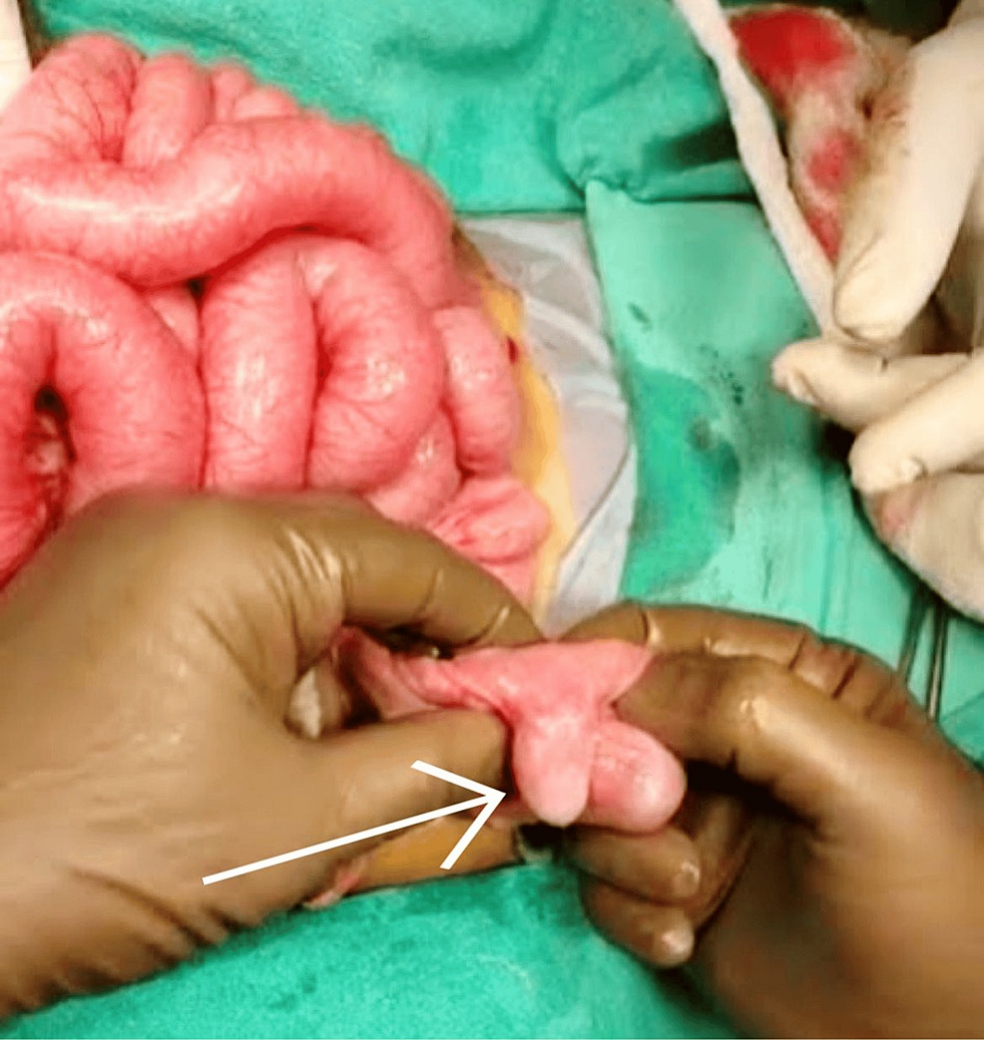
A diverticulum (marked with a white arrow) noted following the reduction of the intussusception, unveiled as the instigator behind this medical crisis

Milking of the bowel was performed, and the intussusception was successfully reduced. The MD was identified as protruding 2 cm inside the proximal ileum, serving as a lead point (Figure 5).

**Figure 6:**
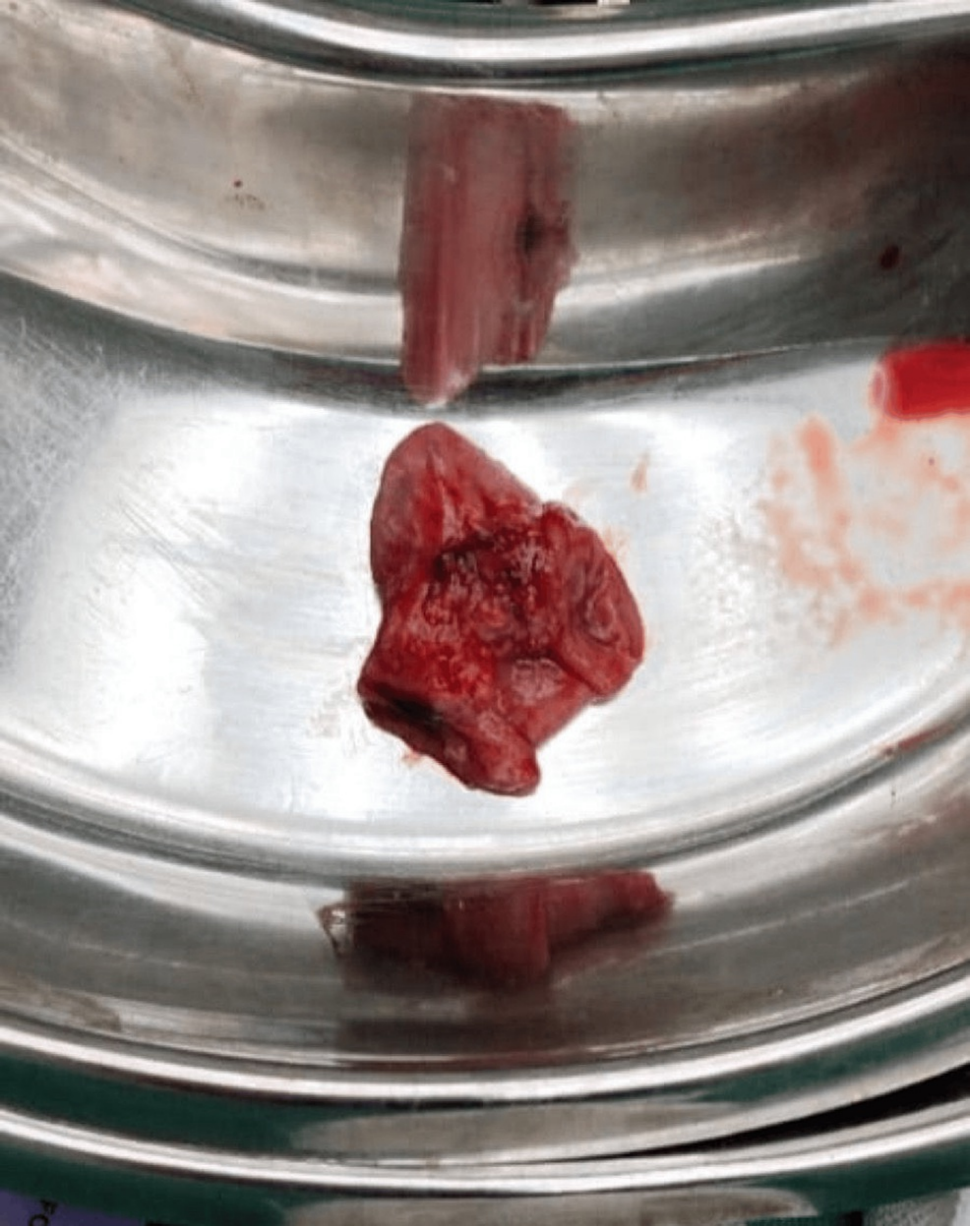
Resected specimen – a segment of the ileum enmeshed with the sinister diverticulum

**Figure 7:**
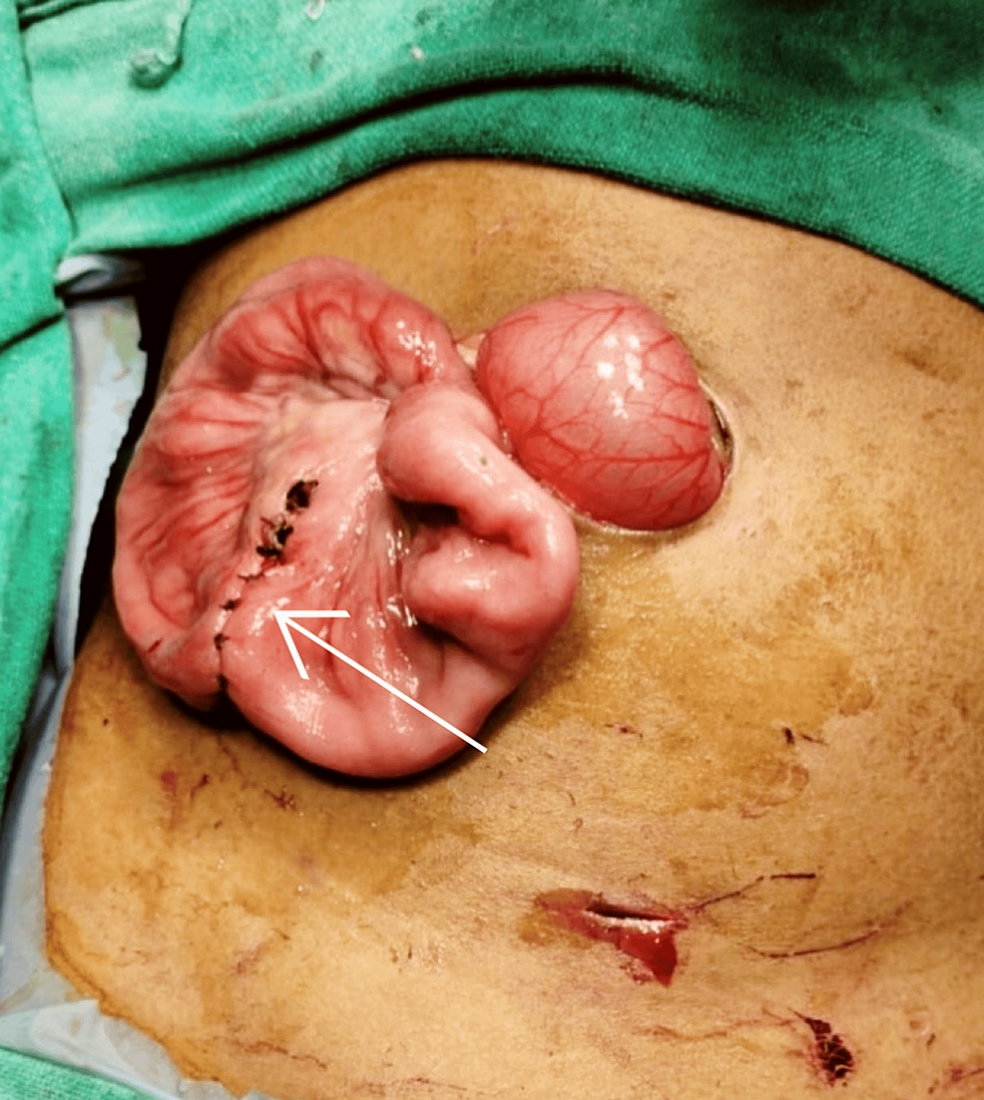
Resection of the affected ileal bowel loop intertwined with the diverticulum, managed with an end-to-end ileoileal anastomosis (marked with a white arrow)

Decompression was done through the milking of the bowel. A 5-cm segment of MD was resected, followed by the completion of an end-to-end ileoileal anastomosis using Vicryl 4-RB sutures (Figure 6, Figure 7).

**Figure 8:**
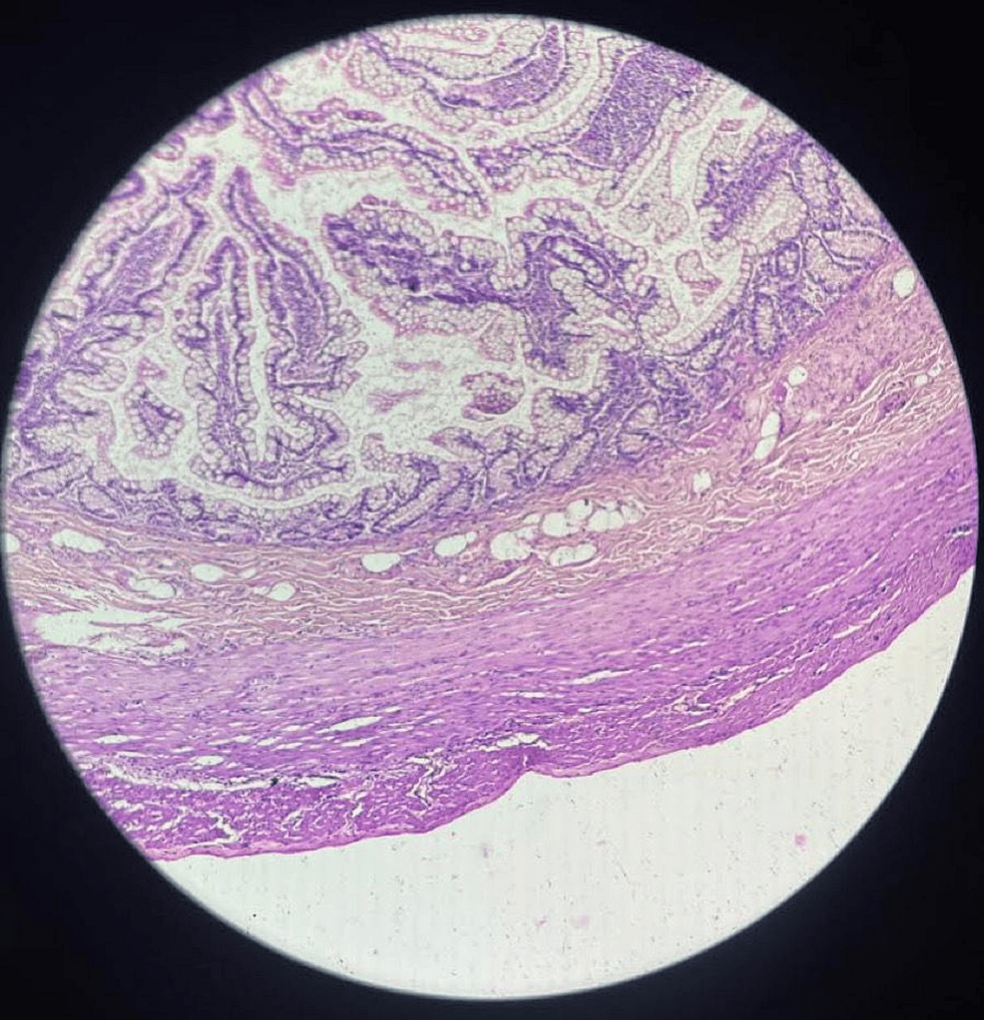
Histopathology showing the gastric mucosa, infiltrated by eosinophils amid areas of congestion, and a sparse presence of polymorphs

A thorough abdominal wash was given. Hemostasis was confirmed. An abdominal drain 20 was inserted and fixed with Silk 2-0 RC. Abdominal closure was done in layers. Rectus closure was performed with Vicryl 3-0 RB. Skin closure was also completed. There were no postoperative complications, and the patient was discharged on day 8. The resected specimen was sent for histopathology. Histopathological analysis revealed single, irregular, grayish-yellow tissue pieces measuring 4 × 3 × 1.5 cm. An outpouching measuring 2 cm was identified. Sections from the proximal and distal margins were unremarkable. Sections from the intussuscepted area showed infiltration of eosinophils, edema, foci of congestion, and scanty polymorphs otherwise unremarkable on histopathology (Figure 8).

The patient remained vitally stable during the one-month follow-up, passing stools normally and maintaining a normal sedentary lifestyle. Follow-up appointments were scheduled monthly for the next six months, during which the patient was observed to be stable, accepting all oral feeds, and passing stools without any active complaints.

## Discussion

Clinically, every child with painless rectal bleeding under the age of two should be suspected of having MD. About half of all lower gastrointestinal bleeding in children under the age of two is caused by it [11]. It has a 1-3% incidence reported and observed as a remnant of the vitelline duct [3]. Other major causes reported for per rectal bleeding are colitis (ulcerative, infectious, and nonspecific), colorectal polyps, hemorrhoids, intussusception, and inflammatory bowel disease [12]. Abdominal pain, gastrointestinal bleeding, and anemia are common symptoms observed in MD, often accompanied by complications such as intussusception, intestinal obstruction, and, infrequently, hernia or perforation [1,3,13,14]. MD-related intestinal obstruction is the cause of 20-25% of the presenting cases [13]. In adults, intestinal obstruction is the most prevalent manifestation, accounting for approximately 40% of cases with symptoms. With MD serving as the leading point, intussusception, a mechanical volvulus of the small intestine centered on a persistent fibrous band that connects the diverticulum to the umbilicus, is the most frequent cause of obstruction. It has been discovered that obstruction happens more frequently in the big MDs [15,16]. According to reports, gastrointestinal bleeding occurs in 25.3% of instances with symptomatic MD in children, obstruction occurs in 46.7% of cases, and inflammation occurs in 19.5% of cases. In both adults and children, intraperitoneal bleeding due to MD is exceedingly uncommon [17]. In an inverted MD, a possible cause might be an irregular peristalsis in the bowel segment immediately adjacent to the diverticulum. Moreover, intussusception in these patients is facilitated by the inverted diverticulum, which can impede bowel function and result in obstruction [18]. There are some research studies reporting that MD increases the likelihood of an inversion since it is not attached to the mesentery or the intestine [3].

## Conclusions

Prompt recognition and intervention are crucial in the gastrointestinal emergencies associated with MD. The patients’ presentation with symptoms of obstruction can be helpful in the diagnosis of ileoileal intussusception, which necessitates urgent surgical intervention. Exploratory laparotomy was found to be helpful in the reduction of intussusception, with the identification and resection of MD acting as the lead point. The rarity of an inverted MD further complicates its clinical recognition, coupled with its association with intussusception, which contributes to the complexity of diagnosis and necessitates timely intervention to prevent serious consequences like bowel ischemia, necrosis, perforation, peritonitis, and sepsis. This case report serves as a stark reminder of the necessity for immediate diagnosis, vigilant monitoring, and swift intervention in gastrointestinal emergencies associated with MD.
